# High-risk T1 esophageal adenocarcinoma in a cervical gastric inlet patch

**DOI:** 10.1055/a-2936-2513

**Published:** 2026-09-01

**Authors:** Augustin Carton, Veronique Van der Voort, Thomas Botelberge, Olivier Plomteux, Noella Blétard, Jérémie Jacques, Philippe Leclercq

**Affiliations:** 1 Department of Gastroenterology and HepatologyClinique CHC MontLégiaLiègeBelgium; 2 Department of Gastroenterology and HepatologyCentre Hospitalier Universitaire (CHU) de LimogesLimogesFrance; 3 Department of Gastroenterology and HepatologyZiekenhuis aan de Stroom (ZAS)AntwerpBelgium; 4 Department of PathologyClinique CHC MontLégiaLiègeBelgium; 5 Department of Gastroenterology and Hepatology 60182 University Hospitals LeuvenLeuvenBelgium

**Fig. 1:**
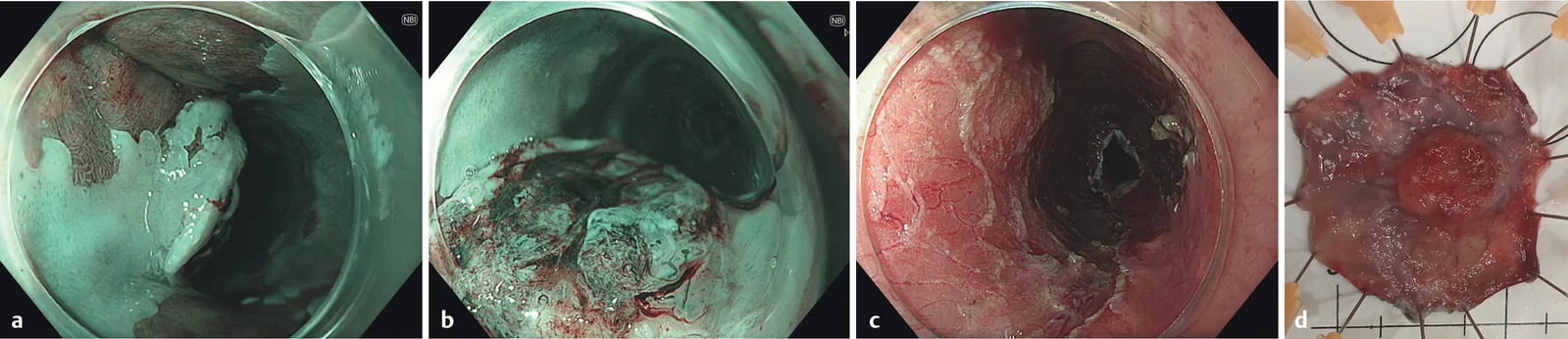
Endoscopic findings of adenocarcinoma arising from ectopic gastric mucosa in the cervical esophagus. ( **a** ) An endoscopic view of the lesion. ( **b)** Narrow-band imaging (NBI) shows distorted glandular and vascular patterns, friability and spontaneous bleeding. ( **c** ) Resection ulcer in the proximal esophagus after ESD. ( **d** ) A resection specimen with a lesion of 20 × 15 mm.

A 60-year-old man was referred for the evaluation of dysphagia with an upper endoscopy. A 20×15 mm Paris-Is sessile lesion was identified on a gastric inlet patch in the cervical esophagus. Narrow-band imaging showed distorted glandular and vascular patterns with friability and spontaneous bleeding, raising the suspicion of superficial adenocarcinoma. Based on these endoscopic findings, the lesion was considered suitable for en-bloc endoscopic resection ( Video 1 ; Fig. 1a, b ).

**Video 1** Endoscopic submucosal dissection of esophageal adenocarcinoma in a cervical gastric inlet patch. Video URI:


The lesion was removed en-bloc by endoscopic submucosal dissection (ESD) under saline immersion, using a clip-and-line traction combined with the tunnelling technique 1​ ( Fig. 1c, d **)** .

**Fig. 2:**
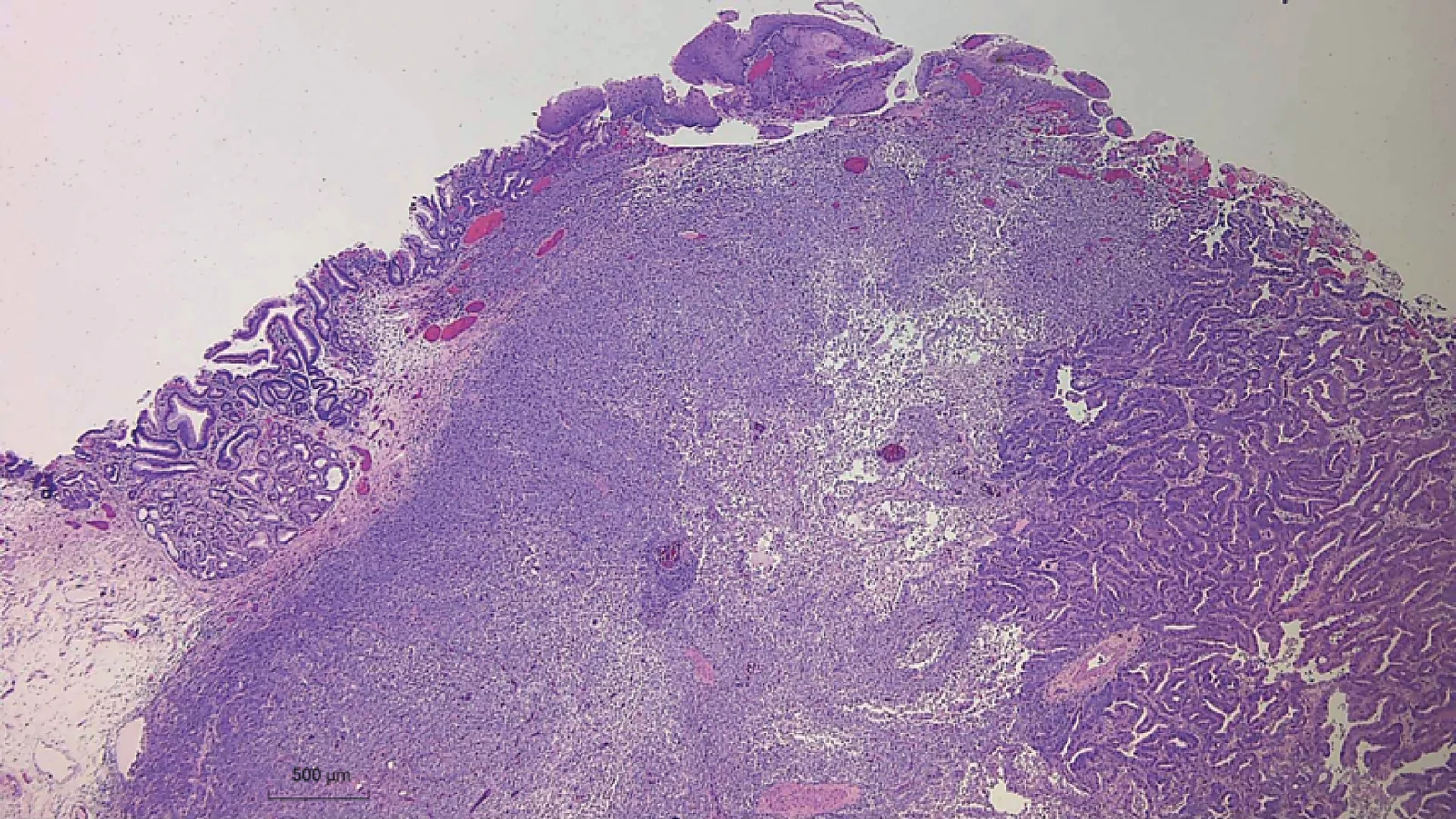
A histological image of the pT1b G3 Nx Mx L1 V0 Pn0 R0 (HM0, VM0) adenocarcinoma, arising from ectopic gastric mucosa.

Histology showed an R0 resection of a poorly differentiated adenocarcinoma (G3) with a submucosal invasion depth of 1,200 µm (pT1b, sm2/3), lymphatic invasion (L1), without venous or perineural invasion (V0, Pn0; Fig. 2 ).

A mild post-ESD stricture was effectively managed endoscopically with two sessions of hydrostatic balloon dilatation.

After multidisciplinary team discussion and shared decision-making, close endoscopic and imaging (endoscopic ultrasonography/positron emission tomography–computed tomography) follow-up was preferred over surgery or additional chemoradiotherapy, with favorable oncological and functional outcomes at 2 years of follow-up.

High-risk T1 esophageal adenocarcinoma (HR-T1 EAC), characterized by submucosal invasion, poor differentiation, and/or lympho-vascular invasion, has traditionally been treated by esophagectomy because of the presumed high risk of lymph node metastasis, up to 46%. 2​ However, ESD enables radical endoscopic resection, and at the same time, more recent studies suggest that the actual risk of lymph node metastasis may be lower than previously thought, potentially below 24%. 2​

As a result, the therapeutic approach to these tumors is currently being re-evaluated. 2​ ​3​

Ongoing research will clarify optimal management, but active surveillance after radical endoscopic resection of HR-T1 EAC may be a reasonable alternative in selected patients, balancing surgical risk and patient preference within a multidisciplinary setting. 4​

Although gastric inlet patches are relatively common, with a reported prevalence ranging from 0.1% to 10% depending on the endoscopic detection method, malignant transformation appears to be rare. 5​ Only isolated cases and small case series of adenocarcinoma arising from gastric inlet patches have been reported in the literature.

This case demonstrates the feasibility of ESD for HR-T1 EAC arising from a gastric inlet patch, supporting its management as esophageal adenocarcinoma even in high-risk disease.

Endoscopy_UCTN_Code_TTT_1AO_2AG_3AD
